# Unraveling Asian Soybean Rust metabolomics using mass spectrometry and Molecular Networking approach

**DOI:** 10.1038/s41598-019-56782-4

**Published:** 2020-01-10

**Authors:** Evandro Silva, José Perez da Graça, Carla Porto, Rodolpho Martin do Prado, Clara Beatriz Hoffmann-Campo, Mauricio Conrado Meyer, Estela de Oliveira Nunes, Eduardo Jorge Pilau

**Affiliations:** 1 Laboratory of Biomolecules and Mass Spectrometry, Department of Chemistry, State University of Maringá, 5790, Colombo Av, Maringá, PR 87020-080 Brazil; 2 Master in Science, Technology and Food Safety, Cesumar Institute of Science, Technology and Innovation – ICETI, University Center of Maringá - UNICESUMAR, 1610, Guedner Av, Maringá, PR 87050-900 Brazil; 3 Department of Animal Science, State University of Maringá, 5790, Colombo Av, Maringá, PR 87020-080 Brazil; 4 Brazilian Agricultural Research Corporation Soybean, Carlos João Strass Rd, Londrina, PR 86001-970 Brazil; 5 Brazilian Agricultural Research Corporation Swine and Poultry, BR-153, Km 110 Distrito de Tamanduá, SC 89715-899 Brazil

**Keywords:** Metabolic pathways, Metabolomics

## Abstract

Asian Soybean Rust (ASR), caused by the biotrophic fungus *Phakopsora pachyrhizi*, is a devastating disease with an estimated crop yield loss of up to 90%. Yet, there is a nerf of information on the metabolic response of soybean plants to the pathogen Untargeted metabolomics and Global Natural Products Social Molecular Networking platform approach was used to explore soybean metabolome modulation to *P. pachyrhizi* infection. Soybean plants susceptible to ASR was inoculated with *P. pachyrhizi* spore suspension and non-inoculated plants were used as controls. Leaves from both groups were collected 14 days post-inoculation and extracted using different extractor solvent mixtures. The extracts were analyzed on an ultra-high performance liquid chromatography system coupled to high-definition electrospray ionization-mass spectrometry. There was a significant production of defense secondary metabolites (phenylpropanoids, terpenoids and flavonoids) when *P. pachyrhizi* infected soybean plants, such as putatively identified liquiritigenin, coumestrol, formononetin, pisatin, medicarpin, biochanin A, glyoceollidin I, glyoceollidin II, glyoceollin I, glyoceolidin II, glyoceolidin III, glyoceolidin IV, glyoceolidin VI. Primary metabolites (amino acids, peptides and lipids) also were putatively identified. This is the first report using untargeted metabolomics and GNPS-Molecular Networking approach to explore ASR in soybean plants. Our data provide insights into the potential role of some metabolites in the plant resistance to ASR, which could result in the development of resistant genotypes of soybean to *P. pachyrhizi*, and effective and specific products against the pathogen.

## Introduction

The soybean (*Glycine max* (L.) Merrill) is prominent among crops due to its agro-economic and nutritional value, used mainly as a source of proteins and oils for human consumption, in animal feeds and for biofuel production^1,2^. However, estimates indicate a necessity to double global agricultural output by 2050 to feed a rising population, hence soybean productivity needs to be increased by 2.4% per annum^3^. Yet, soybean diseases were estimated to reduce crop yield by 11% in the United States^4^ and 50% or greater in the southeastern United States^5^.

The Asian Soybean Rust (ASR) was registered for the first time in Brazil during the 2001/2002 harvest^6^. Recently, Brazilian soybean farmers spent US$ 2.16 billion with fungicides during the 2016/2017 harvest, and 96% of the sum was used for ASR control. Another US$ 1.62 billion was invested on insecticides, totaling US$ 3.78 billion. This amount represented 12.4% of the production costs for the harvest^7^. Despite increasing costs, disease management practices are needed as yield losses of up to 90% have already been reported when control measures were absent^4^.

The fungus *Phakopsora pachyrhizi* is the causal agent of the ASR. The phytopathogen *P. pachyrhizi* survives all-year-round whenever a soybean host plant is available and when conditions favor soybean development, thus supporting epidemic outbreaks^8^. Fungicides, such as demethylation and quinone outside inhibitors are largely used to prevent infection by *P. pachyrhizi*^9^. However, the Fungicide Resistance Action Committee (FRAC) recently reported regions in Brazil with intensive use of chemical fungicides where the efficiency of those products was reduced^10^, which can be caused by intensive use of a single chemical and excessive application. Management strategies to address ASR can range from planting early ripening varieties, field monitoring, eliminating secondary hosts, and even imposing soybean-free growth periods (60–90 days) in the threatened areas^4,9^. Thus, no single solution is available to address *P. pachyrhizi* infection. Therefore, the use of several management strategies must be associated to reduce yield losses and to ensure the crop sustainability, which is time and resource consuming.

Perception of plants by invaders rapidly activates ion flow mechanisms; modulates the production of reactive oxygen species (ROS), primary metabolite modification, and biosynthesis of secondary metabolites; and favors expression of defense genes^11,12^. The signaling cascade can be mediated by plant hormones such as salicylic and jasmonic acid, and ethylene. These hormones can then generate secondary metabolites with antimicrobial properties and modulate plant defenses against pathogens^13–16^. During a pathogen attack, plants recognize pathogens or microbe-associated molecular patterns (PAMPs) and increase its immune defense systems^17^. Nonetheless, some pathogens can reduce plant immunity^18^, which triggers a second immune system known as effector-triggered (ETI). The ETI is activated by the recognition of microbial molecules from cell-surface-membrane proteins^19^. However, *P. pachyrhizi* can bypass both mechanisms and alter the host metabolism^20^.

The vast majority of studies exploring plant metabolites have focused on the prospection of bioactive metabolites. However, with recent advances in analytical methods and annotation tools, metabolic modulation in plants as a result of abiotic and biotic stresses can now be systematically addressed^21^. Untargeted metabolomics has already been employed to improve our understanding of the metabolism of inter-kingdom interactions, including plant-pathogen interactions^22^. The analysis of the whole metabolic profile of a raw plant extract is challenging due to the diversity of chemical classes^23^. Therefore, high efficiency liquid chromatography coupled to mass spectrometry can be used to explore complex matrix, allowing separation and detection of metabolites in a single chromatographic analysis^24^. In addition, tandem mass spectrometry, such as the Q-TOF (Quadrupole - Time of Flight) geometry, enables sequential experiments (MS/MS) to provide more discriminating data for the metabolic identification process^25^.

Metabolomics analysis of complex samples by liquid chromatography - mass spectrometry (LC-MS/MS) generate a large amount of data and the identification process is challenging^26,27^. Therefore, the association of analytical techniques with molecular annotation tools is essential during the identification stage of the vast metabolic chemical diversity present in biological systems. Recently, Dorrestein and colleagues introduced a tandem mass spectrometry (MS/MS) data organizational approach, the Molecular Networking^28,29^. Chemical entities can be arranged, visualized, and compared to databases available in the literature^28–30^. Furthermore, fragmentation patterns similarities can be explored, thus enabling the discovery of novel compounds. Molecular Networking has led to the development of Global Natural Products Social Molecular Networking (GNPS) (http://gnps.ucsd.edu), a data-sharing platform that allows the research community to perform data-driven, crowd-sourced analysis and, it has been widely used for the annotation of MS-based chemical signatures^30^.

In this context, the objective of this study was to use untargeted metabolomics approach to evaluate differences in the metabolic profile of soybean leaves when *P. pachyrhizi* were inoculated or not, and to identify metabolites actively produced by the soybean plant to cope with pathogen infection.

## Materials and Methods

### Plant materials and growth conditions

The genotype PI636463 (susceptible to ASR), provided by the Germplasm Active Bank of Embrapa Soybean was used. The get seeds (daughters seeds) having homogeneous physiological characteristics were obtained from a single seed (mother seed). The experiment using the get seeds was conducted in a greenhouse under the following growth conditions: temperature = 28 ± 2 °C; ultraviolet radiation = 70%, ±10%; and 12 h/12 h light/dark photoperiod, up to vegetative stage V6-V7^31^. Two daughter seeds were sown per pot (5 L) containing substrate (soil, sand and compost; 3:2:2).

### Inoculum preparation

The spores of *P. pachyrhizi* were supplied by the Embrapa Soybean Phytopathology Laboratory (Londrina, Parana, Brazil), obtained from cultivar BRS 284 (susceptible standard), with 92% of germination viability. A total of 500 mL of a spore suspension (3.6 × 10^4^ U mL^−1^) was prepared with sterile water as a vehicle to be used as an inoculant. 250 μL of 70% ethanol solution was added into the suspension as the dispersing agent. For the control group (false-inoculated plants), inoculation with the same excipients was carried out, however, without the presence of spores.

### Experimental design and metabolites extraction

The plants were divided into two groups: (I) control (non-inoculated) and (II) inoculated with *P. pachyrhizi*. The inoculum suspension containing *P. pachyrhizi* spores was distributed in each plant individually using a spray bottle for group II, and without spores for group I. Both groups were submitted to automatic nebulization (40 seconds) every two hours for a period of 14 hours to ensure increased levels of humidity. Increased humidity and absence of light simulates the dew or night breeze conditions of the field, which favors spore germination. After nebulization, control plants were transferred to another greenhouse kept under similar conditions of temperature, relative humidity and photoperiod to avoid contamination. After 14 days, it was possible to visually observe the sporulation of the pathogen and formation of lesions on soybean leaves (data not shown). Thus, leaves from groups I and II were collected, wrapped in aluminum foil, immediately immersed in liquid nitrogen and transported to the laboratory. The samples were stored at −80 °C until metabolites extraction.

### Metabolite extraction procedures

The solvent mixtures ternary 1 (3:1:1; chloroform:methanol:water; v-v), ternary 2 (4:4:2; methanol:acetonitrile:water; v-v) and binary (8:2; methanol:water; v-v) were used to evaluate the fingerprint of soybean leaves from both groups^32^. Control and inoculated soybean leaves (5 whole leaves) were macerated separately under liquid nitrogen. Then, about 100 mg of plant material was transferred to 2 ml glass vials and 1.5 ml of the solvent mixture was added. The solution was shaken in vortex for 10 seconds (Vortex-Genie 2, Scientific Industries, New York, USA) and submitted to ultrasound (USC-1400, Unique, Brazil) for 25 minutes. The solution was centrifuged at 12,000 rpm for 10 minutes at 4 °C. The supernatant was collected and filtered using a 0.22 μm PVDF syringe filter (Millex-GV Durapore). The extraction procedures were performed in three replicates for each solvent system and for each group (I and II). The samples were concentrated in nitrogen flow and stored at −80 °C until further analysis.

### LC-MS/MS analysis

Extracts were resuspended in 600 μl water:acetonitrile (1:1; v-v) and 3 μL of each extract were injected and analyzed ultra-high performance liquid chromatograph (Shimadzu, Nexera X2, Japan) coupled to a hybrid quadrupole time-of-flight high resolution mass spectrometer (Impact II, Bruker Daltonics Corporation, Germany) equipped with an electrospray ionization source. Chromatographic separation was performed using an Acquity UPLC HSS T3 C18 column (Waters, USA, 1.7 μm, 2.1 × 100 mm) at flow rate of 0.250 mL min^−1^ ^33^. The gradient mixture of solvents A (H_2_O with 0.1% formic acid, v:v) and B (acetonitrile with 0.1% formic acid, v-v) was as follow: 5% B 0–1 min, 70% B 1–10 min, 98% B 12–20 min and maintained at 5% B 20–25 min at 40 °C, the final five minutes being intended for reconstitution of the column for the next analysis. The instrument was calibrated using a solution of sodium formate (10 mmol L^−1^; isopropanol:water; 1:1; v-v) containing 50 µL concentrated formic acid. The ionization source was operated in the positive ionization mode and adjusted to 4500 V, with a potential plate end of −500 V. The dry gas parameters were set to 8 L min^−1^ at 180 °C with a nebulization gas pressure of 4 bar. The data were obtained in a range of *m/z* 50 to 1800 with an acquisition rate of 5 Hz. The 5 most intense ions were selected for automatic fragmentation (AutoMS/MS). The data were acquired by the Hystar Application software version 3.2 and Otof Control (Bruker Daltonics Corporation, Germany) and were converted to the mzXML file format^34^.

### Molecular networking

The MS/MS data was transferred to the GNPS Molecular Networking server to generate the chemical map (ID = e26e17c3ffca42dc9968f260c2b01cee), according to the GNPS documentation^30^. Solvent and injection blanks were subtracted to generate the chemical map. The molecular network (MN) was generated so that the mass tolerance of precursor ions was 0.02 Da, as this value influences the clustering of almost identical fragmentation spectra (MS/MS). The mass variances of ion fragments for each group of acquired MS/MS spectra were stipulated as ±0.02 Da for clustering (consensus spectrum creation). The lines (connections between nodes) were formed only if the cosine score was above 0.7 and with a minimum correspondence of 4 peaks in the fragmentation spectrum. The MN spectra were then compared to the spectra of the GNPS spectral libraries, such as ReSpect, Massbank and HMDB^35–37^ where the same data parameters were applied to the sample spectra. MN data were visualized in the Cytoscape software^38^. Ion fragmentation spectra with similarities to the mass spectral libraries had the fragmentation spectra manually verified and the mass error calculated, using a mass error tolerance of less than 10 ppm. Area-proportional Venn diagrams from exported molecular features data using GNPS were constructed using meta-chart (https://www.meta-chart.com/).

## Results

### Solvent effect on metabolites extraction

**Figure 1:**
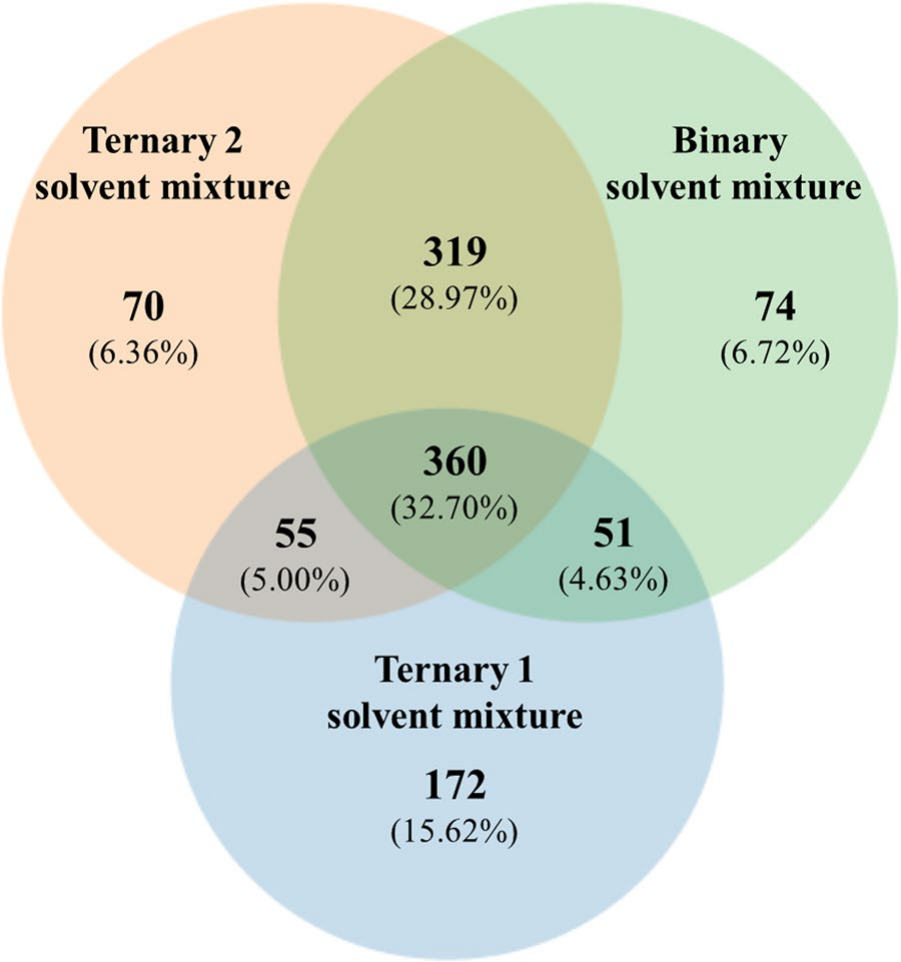
Venn diagram showing the distribution of chemical entities extracted from soybean control leaves using ternary solvent mixture 1 (3:1:1 chloroform: methanol: water; v-v), ternary solvent mixture 2 (4:4:2 methanol: acetonitrile: water; v-v) and binary solvent mixture (8:2 methanol: water; v-v). Chemical entities were detected using an UHPLC-ESI(+)-MS/MS.

**Figure 2:**
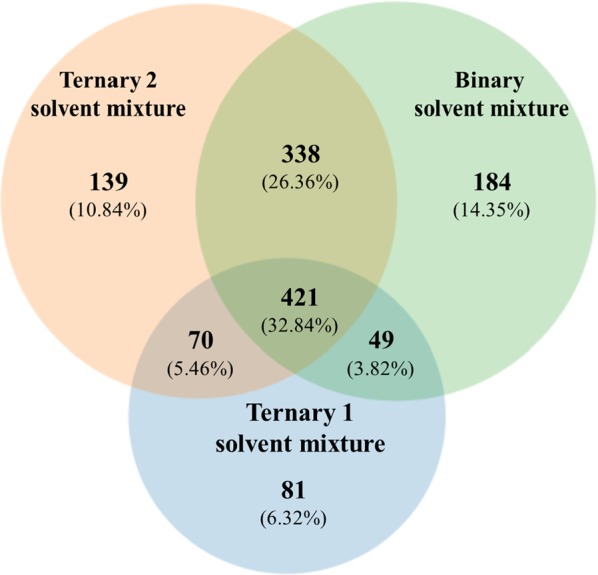
Venn diagram showing the distribution of chemical entities extracted from soybean leaves inoculated with *P. pachyrhizi*, using ternary solvent mixture 1 (3:1:1 chloroform: methanol: water; v-v), ternary solvent mixture 2 (4:4:2 methanol: acetonitrile: water; v-v) and binary solvent mixture (8:2 methanol: water; v-v). Chemical entities were detected using an UHPLC-ESI(+)-MS/MS.

Extraction procedures varying in solvents were used to obtain abundant diversity of metabolites from both groups. Quantities of chemical entities extracted from control (group I) soybean leaves or when soybean leaves were inoculated with *P. pachyrhizi* (group II) using extraction solvent mixtures ternary 1, ternary 2, and binary were compiled in Venn diagrams (Figs. 1 and 2, respectively).

A total of 1101 chemical entities were observed in group I (Fig. 1). Of those, 360 chemical entities were extracted using the three extractor solvent mixtures, which represents 32.70% of the total (Fig. 1). The ternary 2 and binary solvent mixtures resulted in the highest quantities of detected chemical entities, 804 for each solvent mixture (73.02% of total). The ternary 1 solvent mixture resulted in the lowest quantity of detected chemical entities, 638 (57.95% of total). However, ternary 1 solvent mixture had the highest amount of unique chemical entities, 172 (15.62% of total; Fig. 1).

When soybean leaves were inoculated with *P. pachyrhizi* (group II), a total of 1282 chemical entities were observed (Fig. 2). This amount represented an increase of 14.12% when compared to group I. Of the total, 421 (32.84% of total) were extracted using the three extractor solvent mixtures (Fig. 2). The ternary 2 and binary solvent mixtures extracted the highest quantity of chemical entities, 968 (75.51% of total) and 992 (77.38% of total) respectively. The binary, ternary 2 and ternary 1 solvent mixture extracted 184 (14.35% of total), 139 (10.84% of total) and 81 (6.32% of total) unique chemical entities, respectively (Fig. 2).

### Molecular networking and metabolites identification

**Figure 3:**
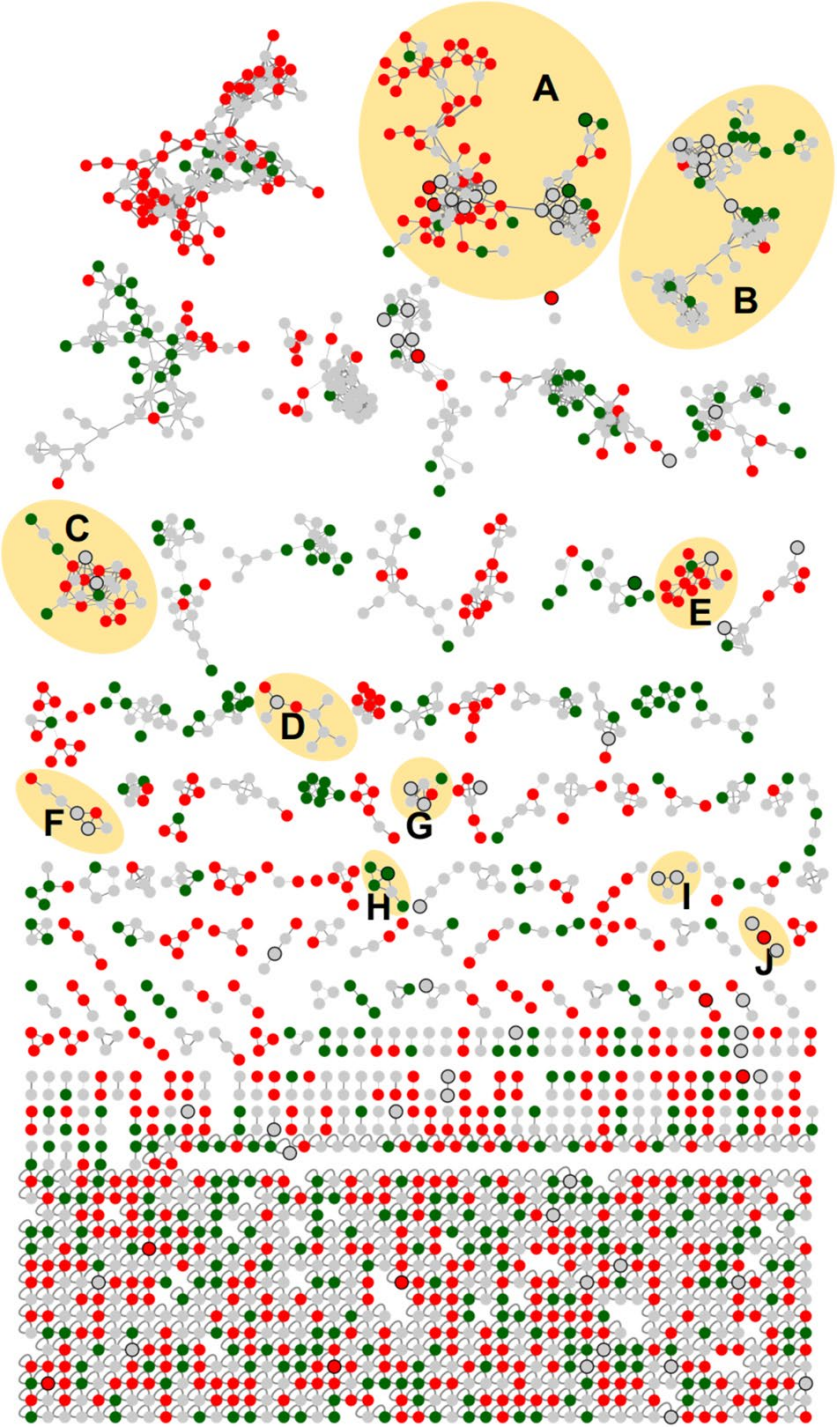
Molecular Network of the MS/MS spectra obtained by the analysis of the soybean control leaves, or inoculated with *P. pachyrhizi*, using solvent mixtures ternary 1 (3:1:1 chloroform: methanol: water; v-v), ternary 2 (4:4:2 methanol: acetonitrile: water; v-v) and binary (8:2 methanol: water; v-v). Green nodes correspond to the MS/MS spectra of soybean control leaves samples. Red nodes correspond to the MS/MS spectra of soybean leaves inoculated with *P. pachyrhizi*. Gray nodes correspond to the MS/MS spectra shared spectra in both samples. The edge width represents the cosine score (0.7 to 1.0). The black bold borders nodes represent the MS/MS spectra that had hits with the spectra of the GNPS libraries. (**A**) flavonoids/isoflavonoids, (**B**) saponins, (**C**) lipids, (**D**) amino acids, (**E**) flavonoids/isoflavonoids, (**F**) flavonoids (**G**) lipids, (**H**) chlorophyll, (**I**) carboxylic acids, (**J**) peptides.

The manual evaluation of each chemical entity detected by a mass spectrometer using an untargeted metabolomics approach is a challenging task. Thus, Molecular Networking tool was used to organize the MS/MS data (Fig. 3). Each node represents a chemical entity, which can be connected to other nodes according to chemical structure similarities. A node identified using the GNPS or open access libraries can then be used to identify another node in the same cluster by extrapolating gain or loss of a chemical group.

When chemical entities of groups I and II were combined, a total of 1917 nodes (consensus spectra) were obtained in the MN (Fig. 3). Of these, 625 consensus spectra (red nodes) were obtained exclusively when *P. pachyrhizi* was inoculated on soybean leaves; 406 consensus spectra (green nodes) were obtained from the control group; and 886 consensus spectra (gray nodes) were obtained by samples of both groups. Fifty-two consensus spectra (black bold border nodes) had hits with the GNPS library (2.71% of the total). Furthermore, the MN had 97 clusters of nodes interconnected. Clusters containing at least one flavonoid/isoflavonoid, saponins, lipid, amino acid, chlorophyll, carboxylic acids or peptides were identified (Fig. 3A–J).

**Figure 4:**
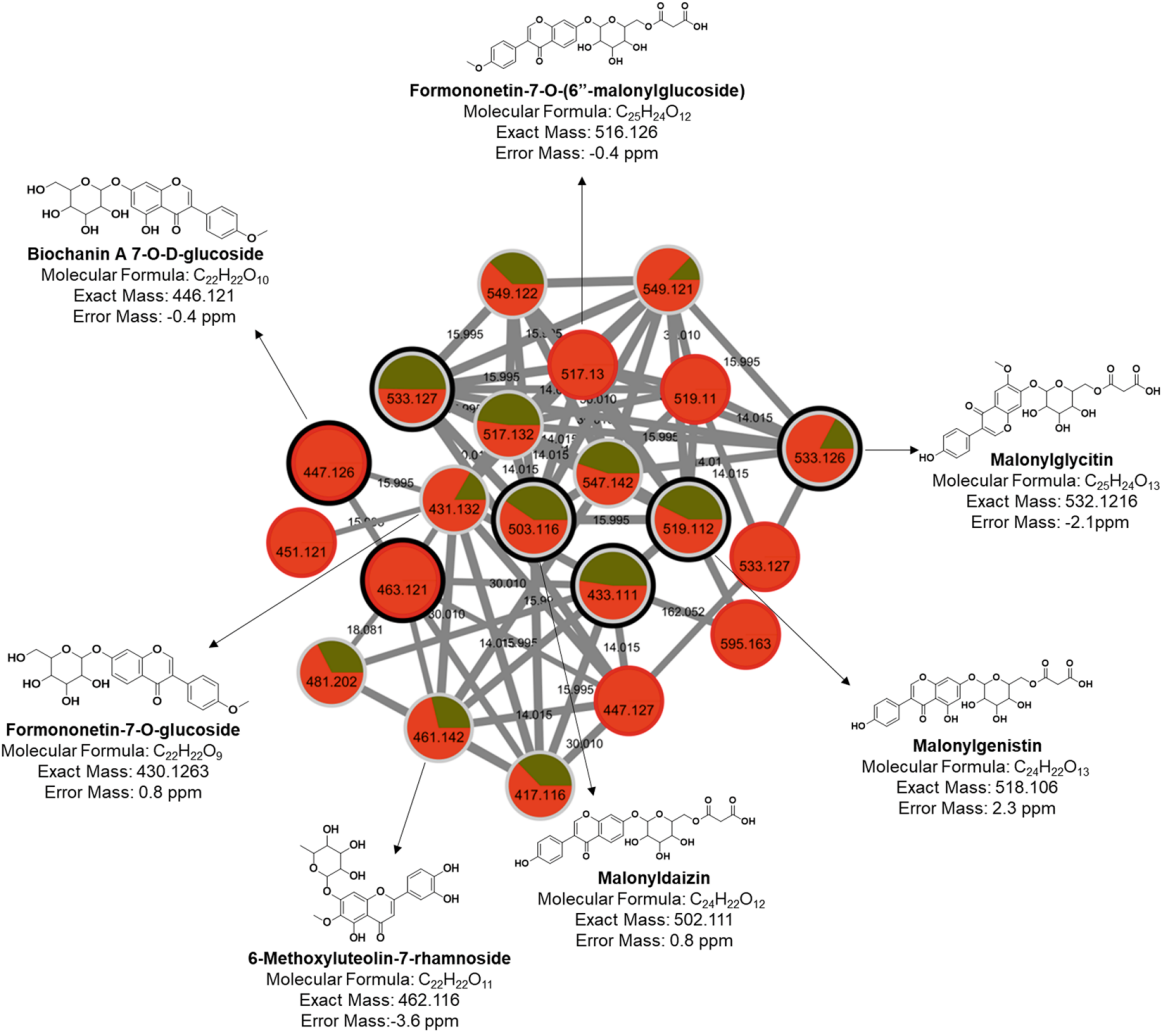
Cluster of isoflavonoids and flavonoids putatively characterized by molecular network obtained from MS/MS data from leaves of control and inoculated with *P. pachyrhizi*. The edge width represents the cosine score (0.88 to 0.99). The edge label represents the mass difference between nodes (14.016 Da, 15.999 Da and 162.052 Da). The black bold borders nodes represent the MS/MS that had hits with the spectra of the GNPS libraries. The pie chart within each node corresponds to the percentage relative of the metabolite in the sample, green indicates soybean control leaves, and red when soybean leaves were inoculated with *P. pachyrhizi*.

Metabolites putatively identification was confirmed manually by verification of fragmentation spectra (data not shown). Mass errors ranged from 0 to 3.9 ppm. Part of one cluster (Fig. 3A) containing the MS/MS spectra of [M + H]^+^ *m/z* 447.125, [M + H]^+^ *m/z* 463.121, [M + H]^+^ *m/z* 503.116 and [M + H]^+^ *m/z* 519.112, putatively identified as biochanin A 7-O-D-glucoside, 6-methoxyluteolin-7-rhamnoside, malonyldaidzin and malonylgenistin, respectively, was explored to highlight the potential of the GNPS approach (Fig. 4). These compounds are classified as isoflavone and flavones. Analogues of some of these compounds were separated by 14.016 Da, 15.999 Da and 162.052 Da, which are attributed to CH_2_, O and C_6_H_10_O_5_ differences, respectively. All the nodes had higher spectral similarity since they were clusterized with relatively high cosine scores (0.88–0.99). Using this approach, other ions belonging to the same cluster could be putatively identified. Furthermore, a pie chart layout^39^ was generated using the peak ion area in each sample group (control and inoculated) for qualitative evaluation.

**Figure 5:**
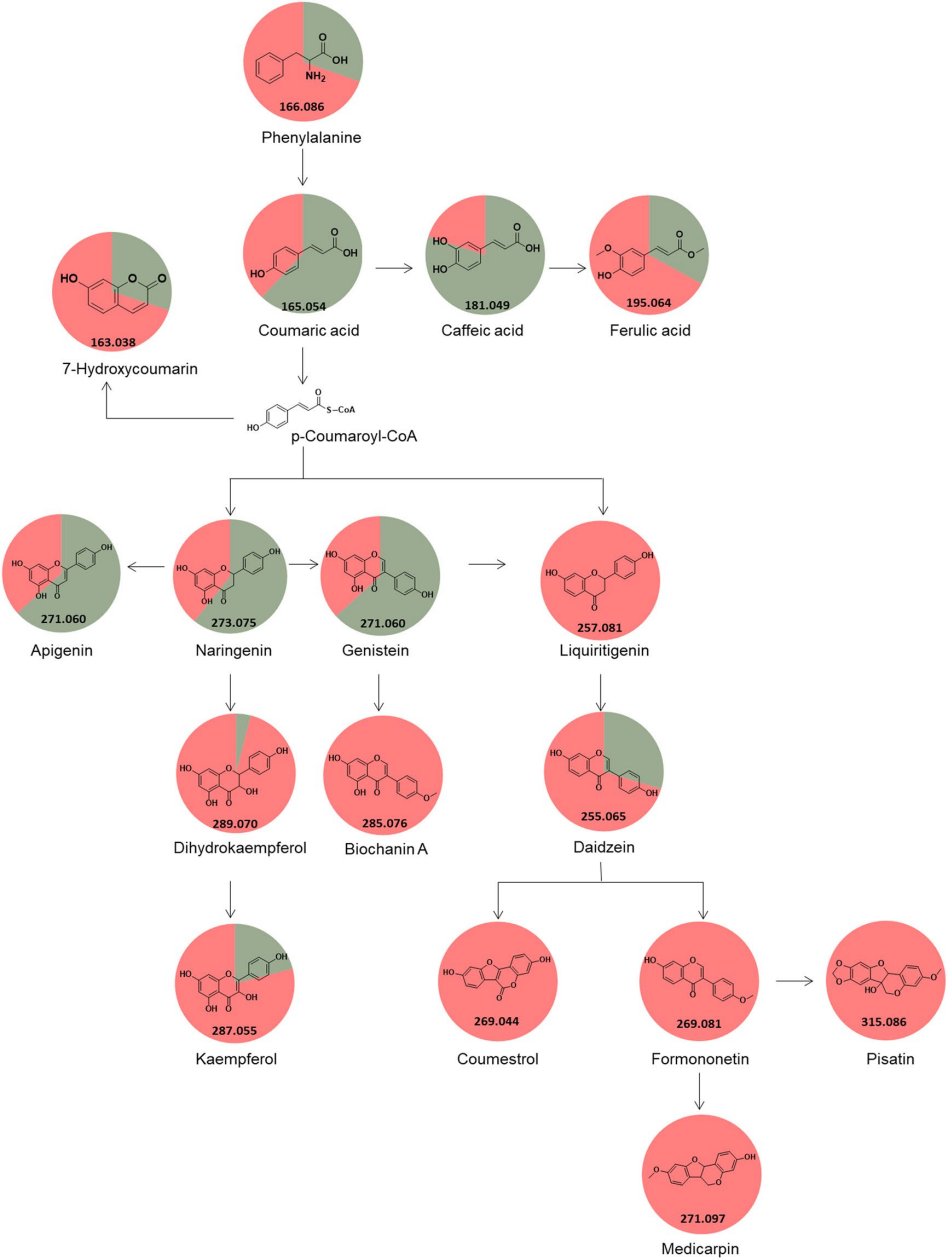
Summary pathway of phenylpropanoid biosynthesis. The pie chart corresponds to the percentage relative of the metabolite in the sample, green indicates soybean control leaves, and red when soybean leaves were inoculated with *P. pachyrhizi*. Adapted from Kanehisa^57^.

**Figure 6:**
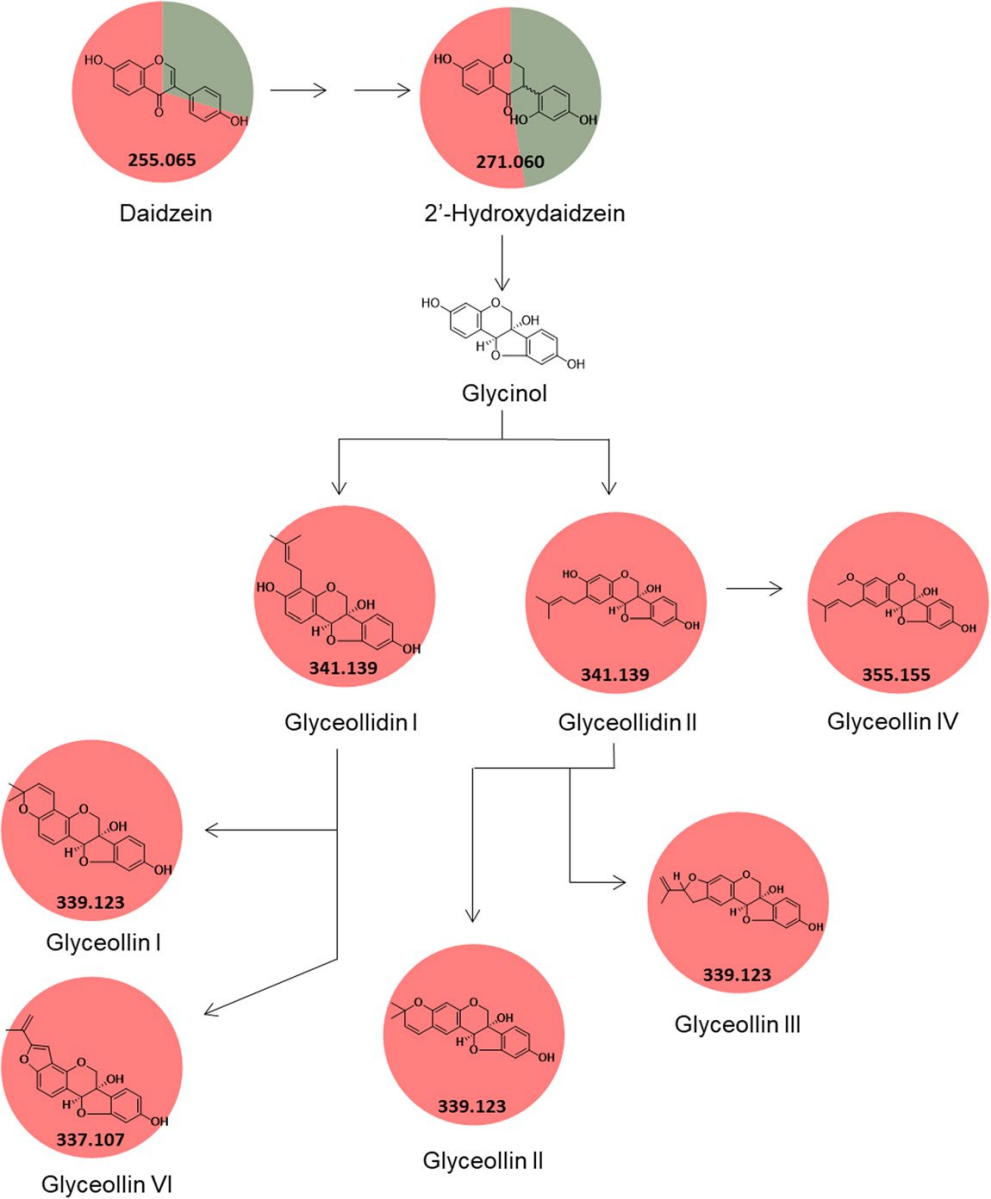
Glyceollin and glyceollidin biosynthesis pathway. The pie chart corresponds to the percentage relative of the metabolite in the sample, green indicates soybean control leaves, and red when soybean leaves were inoculated with *P. pachyrhizi*. Adapted from Simons^58^.

Using the GNPS approach and manual verification of fragmentation spectra, another 94 metabolites were putatively identified (Supplementary Table S1), which represented 4.90% of total. These metabolites belong to the classes of amino acids, nucleoside, vitamin, nucleotide, peptide, phenylpropanoids, flavonoids, isoflavonoids, organic acids, lipids, terpenoids and chlorophyll. From the total, 67 metabolites were identified in both groups (control and inoculated), 26 metabolites were putatively identified exclusively when soybean leaves were inoculated with *P. pachyrhizi*, and one metabolite was putatively identified in the group I. Of the ions exclusively putatively identified in group II, liquiritigenin, coumestrol, formononetin, pisatin, medicarpin, biochanin A (Fig. 5), glyoceollidin I, glyoceollidin II, glyoceollin I, glyoceolidin II, glyoceolidin III, glyoceolidin IV, glyoceolidin VI (Fig. 6) were identified.

## Discussion

As expected, each solvent mixture extracted diverse quantities of unique chemical entities (Figs. 1 and 2). Ternary 2 and binary solvent mixtures, which tends to favor the extraction of polar metabolites, extracted more of the exclusive chemical entities when *P. pachyrhizi* was inoculated (Fig. 2). On the other hand, the ternary 1 solvent mixture, which was used to favor non-polar metabolites, resulted on lowest amount of chemical entities (Fig. 2).

The whole metabolome of a given matrix can be complex, containing compounds with a large of range polarity^40^. Solvent mixtures used as extractors are likely to impact the outcome of a study and should promote reproducible and comprehensive data, thus combinations of solvents were used in this study (ternary 1, ternary 2 and binary solvent mixtures). Using one solvent, or a solvent mixture can be a useful to explore a given sample when there is prior knowledge of the chemical class to be prospected, but with limitations. Conversely, the use of different solvent mixtures is needed to comprehensively explore the chemical ecology of a given matrix. Metabolites which were not known previously can be detected (Figs. 3 and 4), which expands the knowledge and provides novel insights about specific questions. For example, the soybean metabolome modulation was expected when the fungus *P. pachyrhizi*, which is known to cause decreased soybean crop yield^7^, was inoculated in the soybean plant. Indeed, phenylpropanoid, glyceollin and glyceollidin were observed only when *P. pachyrhizi* was inoculated (Figs. 5 and 6). These metabolites are involved in plant metabolism caused by biotic stress, where precursor molecules of biochemical pathways undergo chemical transformations bye enzymes, leading to the production of secondary defense metabolites^41^. Hence, the combination of untargeted metabolomics and with the GNPS was used to prospect possible metabolites involved in the disease modulation.

Several of the identified metabolites are used by plants as defense mechanisms to counter microbial infections, such as terpenes, phenolics and N and S containing compounds^42^. Likewise, amino acids were observed (Supplementary Table S1), and these are important precursors of relevant secondary metabolites pathways that participate in plant defense mechanisms such as salicylic acid, polyamines, alkaloids, phenolic compounds, phenylpropanoids^43^.

In this study, we putatively identified four amino acids present in both groups: proline, phenylalanine, tyrosine and tryptophan. Phenylalanine is one of the main precursors of plant defense metabolites^44^. Phenylalanine is converted to trans-cinnamic acid and ammonia by the enzyme phenylalanine ammonia-lyase (PAL). Trans-cinnamic acid can then be incorporated into several phenolic compounds (4-coumaric acid, caffeic acid, ferulic acid and sinapic acid), which are present in the formation of esters, coumarins, flavonoids and lignins^45^.

The phenylpropanoids are plant secondary metabolites derived from aromatic amino acids. The induction of phenylpropanoid, flavonoid, and isoflavonoid metabolic pathway genes as a defense response to *P. pachyrhizi* was reported in transcriptome studies of susceptible and resistant soybean genotypes^46–48^. We putatively identified 16 phenylpropanoids (Supplementary Table S1). Of those, liquiritigenin, coumestrol, formononetin, pisatin, medicarpin, biochanin A were presented, which are in the pathway of phenylpropanoid biosynthesis, were observed only when *P. pachyrhizi* was inoculated (Fig. 5).

Flavonoids and isoflavonoids are metabolites known as phytoalexins, antimicrobial substances that are synthesized by plants primarily after invasion or contact pathogens occur. Thus, the accumulation of these substances when *P. pachyrhizi* were inoculated soybean leaves may be indicate active plant defense against the pathogen^49^.

Coumestrol, glyceollin and glyceollidin are isoflavonoids, belonging to subclasses of coumestans and pterocarpans, respectively. Both compounds are derived from daidzein (precursor), one of the major isoflavones belonging to soybean grains and leaves. We putatively identified glyoceollidin I, glyoceollidin II, glyoceollin I, glyoceolidin II, glyoceolidin III, glyoceolidin IV, glyoceolidin VI when *P. pachyrhizi* were inoculated soybean leaves (Fig. 6). Simons and co-workers^50^ reported the accumulation of coumestrol, glyceollin and glyceollidin in soybeans grown in the presence of the pathogen *Rhizopus spp*. Likewise, the phytoalexin glyceollin was also only detected when soybean leaves inoculated with *P. pachyrhizi*^51^.

Lipids and fatty acids are essential constituents of plant cells, providing structural integrity and energy for various metabolic processes. They can act as signal transduction mediators, playing both defense and signaling roles in response to biotic and abiotic stresses^52^. Some polyunsaturated fatty acids, such as linoleic and linolenic acids are precursors of oxylipins in plants^53^. Oxylipins correspond to a wide range of products generated by self-oxidation or enzymatic oxidation of polyunsaturated fatty acids. Jasmonic acid was putatively identified for both groups in this study. Considered as a signaling substance, jasmonic acid is able to recognize plant infection by the pathogen and activate biosynthesis of defensive metabolites such as phytoalexins, terpenoids and flavonoids^19^.

Other putatively identified compounds were saponins, which are part of the terpenes class. Saponins can regulate plant growth and defense against insects and pathogens. They are present in high concentration in healthy plants, as they act as barriers to chemical reactions from fungi attack^54^. Indeed, *Avena strigosa* saponin-deficient genotypes had their resistance compromised to a variety of fungal pathogens^55^. The medicagenic acid saponins have been reported to have antifungal activity for *Sclerotium rolfsii*, *Rhizoctonia solani*, *Trichoderma viride*, *Aspergillus niger* and *Fusarium oxysporum*^56^. In this study, this class of compounds was putatively identified in control and inoculated leaves (Supplementary Table S1). However, the importance of saponins when soybean plants are infected by *P. pachyrhizi* is still unknown.

## Conclusions

Asian Soybean Rust is an important disease and efficient pathogen control is challenging. Using untargeted metabolomics, we proposed a novel approach to evaluate the soybean metabolome modulation when affected by the Asian Soybean Rust disease. The combination of mass spectrometry and the Global Natural Products Social Molecular network was successful to identify exclusive metabolites present when soybean leaves were inoculated with *P. pachyrhizi*. Identification of metabolites associated with plant defenses is powerful information on the development of resistant genotype to resist *P. pachyrhizi*, as well as in the development of more effective and specific products against the disease.

## Supplementary information

Supplementary information.
