# Supplementary material for: Unraveling Asian Soybean Rust metabolomics using mass spectrometry and Molecular Networking approach
Source: Sci Rep. 2020 Jan 10;10:138. doi: 10.1038/s41598-019-56782-4 (PMC6954191; doi:10.1038/s41598-019-56782-4)
Supplement: Supplementary file 1 — Supplementary information. [file 41598_2019_56782_MOESM1_ESM.pdf]

Unraveling Asian Soybean Rust metabolomics using mass spectrometry and Molecular Networking approach

Evandro Silva[1], José Perez da Graça[4], Carla Porto[1,2], Rodolpho Martin do Prado[1,3], Clara Beatriz Hoffmann-Campo[4], Mauricio Conrado Meyer[4], Estela de Oliveira Nunes[4], Eduardo Jorge Pilau[1,*]

[1]Laboratory of Biomolecules and Mass Spectrometry, Department of Chemistry, State University of Maringá, 5790, Colombo Av, Maringá, PR, 87020-080, Brazil.

[2]Master in Science, Technology and Food Safety and Cesumar Institute of Science, Technology, and Innovation – ICETI, University Center of Maringá - UNICESUMAR, 1610, Guedner Av, Maringá, PR, 87050-900, Brazil.

[3]Department of Animal Science, State University of Maringá, 5790, Colombo Av, Maringá, PR, 87020-080, Brazil.

[4]Brazilian Agricultural Research Corporation Soybean, Carlos João Strass Rd, Londrina, PR, 86001-970, Brazil.

SUPPORTING INFORMATION

Table S1: Metabolites identified using the molecular networking tool and MS/MS spectra.

Proposed compound identify	Molecular formula	$[M+H]^+$ Measured	$[M+H]^+$ Theoretical	Mass accuracy (ppm)	Leaves Control	Leaves Inoculated
**Amino acid**						
Proline	$C_5H_9NO_2$	116.0706	116.0706	0.0	x	x
Phenylanine	$C_9H_{11}NO_2$	166.0862	166.0862	0.0	x	x
Tyrosine	$C_9H_{11}NO_3$	182.0809	182.0812	-1.6	x	x
Tryptophan	$C_{11}H_{12}N_2O_2$	205.0971	205.0972	-0.5	x	x
**Nucleoside**						
Cytidine	$C_9H_{13}N_3O_5$	244.0927	244.0928	-0.4		x
Uridine	$C_9H_{12}N_2O_6$	245.0766	245.0768	-0.9	x	x
Adenosine	$C_{10}H_{13}N_5O_4$	268.1036	268.1040	-1.6	x	x
**Vitamin**						
Riboflavin	$C_{17}H_{20}N_4O_6$	377.1454	377.1456	-0.4		x
**Nucleotide**						
Adenosine monophosphate	$C_{10}H_{14}N_5O_7P$	348.0698	348.0704	-1.6	x	x
**Peptide**						
Threonylleucine	$C_{10}H_{20}N_2O_4$	233.1499	233.1496	1.3	x	x
Aspartylleucine	$C_{10}H_{18}N_2O_5$	247.1284	247.1288	-1.6	x	x
**Phenylpropanoid**						
Coumaric acid	$C_9H_8O_3$	165.0547	165.0546	0.6	x	x

Caffeic acid	$C_9H_8O_4$	181.0495	181.0495	0.0	x	x
Ferulic acid	$C_{10}H_{10}O_4$	195.0652	195.0652	0.0	x	x
**Coumarin**						
7-Hydroxycoumarin	$C_9H_6O_3$	163.0388	163.0389	-0.6	x	x
7-Methoxycoumarin	$C_{10}H_8O_3$	177.0542	177.0546	-2.3	x	x
**Flavonoid**						
Liquiritigenin	$C_{15}H_{12}O_4$	257.0812	257.0808	1.6		x
Apigenin	$C_{15}H_{10}O_5$	271.0591	271.0601	-3.7	x	x
Kaempferol	$C_{15}H_{10}O_6$	287.0554	287.0550	1.3	x	x
Dihydrokaempferol	$C_{15}H_{12}O_6$	289.0708	289.0707	0.5	x	x
Naringenin-7-O-glucoside	$C_{21}H_{22}O_{10}$	435.1284	435.1286	-0.4		x
Eriodictyol-7-O-glucoside	$C_{21}H_{22}O_{11}$	451.1221	451.1235	-3.1		x
6-Methoxyluteolin-7-rhamnoside	$C_{22}H_{22}O_{11}$	463.1218	463.1235	-3.6		x
Afrormosin 7-O-glucoside	$C_{23}H_{24}O_{10}$	461.1448	461.1442	1.3	x	x
Malonylgenistin	$C_{24}H_{22}O_{13}$	519.1125	519.1113	2.3	x	x
Chrysoeriol 7-O-(6"-malonyl-glucoside)	$C_{25}H_{24}O_{14}$	549.1239	549.1239	0.0	x	x
Kaempferol-3-O-rutinoside	$C_{27}H_{30}O_{15}$	595.1653	595.1657	-0.8	x	x
Rutin	$C_{27}H_{30}O_{16}$	611.1603	611.1607	-0.6	x	x
Robinin	$C_{33}H_{40}O_{19}$	741.2224	741.2237	-1.7	x	x
Quercetin 3-rhamninoside	$C_{33}H_{40}O_{20}$	757.2173	757.2186	-1.7	x	x
Kaempferol 3-sophorotrioside	$C_{33}H_{40}O_{21}$	773.2113	773.2135	-2.8	x	x
Dadzein	$C_{15}H_{10}O_4$	255.0657	255.0652	2.0		x
2′-Hidroxydaidzein	$C_{15}H_{10}O_5$	271.0605	271.0601	1.5	x	x
Glycitein	$C_{16}H_{12}O_5$	285.0764	285.0758	2.3		x

Calycosin	$C_{16}H_{12}O_5$	285.0766	285.0758	3.0		x
Biochanin A	$C_{16}H_{12}O_5$	285.0764	285.0758	2.3		x
Formononetin	$C_{16}H_{12}O_4$	269.0818	269.0808	3.6		x
Daidzin	$C_{21}H_{20}O_9$	417.1183	417.1180	0.7	x	x
Formononetin-7-O-glucoside	$C_{22}H_{22}O_9$	431.1340	431.1337	0.8		x
Biochanin A 7-O-D-glucoside	$C_{22}H_{22}O_{10}$	447.1284	447.1286	-0.4		x
Glycitein 7-O-glucoside	$C_{22}H_{22}O_{10}$	447.1292	447.1286	1.3		x
Malonyldaidzin	$C_{24}H_{22}O_{12}$	503.1188	503.1184	0.8	x	x
Formononetin 7-O-(6"-malonylglucoside)	$C_{25}H_{24}O_{12}$	517.1338	517.1340	-0.4	x	x
Biochanin A 7-O-glucoside-6"-O-malonate	$C_{25}H_{24}O_{13}$	533.1291	533.1289	0.4	x	x
Malonylglycitin	$C_{25}H_{24}O_{14}$	533.1278	533.1289	-2.1	x	x
Coumestrol	$C_{15}H_8O_5$	269.0444	269.0445	-0.2		x
Isotrifoliol	$C_{16}H_{10}O_6$	299.0548	299.0550	-0.7		x
Sojagol	$C_{20}H_{16}O_5$	337.1075	337.1071	1.3	x	x
Medicarpin	$C_{16}H_{14}O_4$	271.0971	271.0964	2.6	x	x
Pisatin	$C_{17}H_{14}O_6$	315.0869	315.0863	1.9		x
Phaseolin	$C_{20}H_{18}O_4$	323.1275	323.1278	-0.9		x
Glyceollin I	$C_{20}H_{18}O_5$	339.1234	339.1227	2.1		x
Glyceollin II	$C_{20}H_{18}O_5$	339.1236	339.1227	2.7		x
Glyceollin III	$C_{20}H_{18}O_5$	339.1223	339.1227	-1.2		x
Glyceollin VI	$C_{20}H_{16}O_5$	337.1068	337.1070	-0.6		x
Glyceollidin I/II	$C_{20}H_{20}O_5$	341.1390	341.1384	1.9		x
Glyceollin IV	$C_{21}H_{22}O_5$	355.1554	355.1540	3.9		x
**Organic Acids**						
Pipecolic acid	$C_6H_{11}NO_2$	130.0861	130.0863	-1.5	x	x

Salicylic acid	$C_7H_6O_3$	139.0391	139.0390	0.7	x	x
Citric acid	$C_6H_8O_7$	193.0341	193.0343	-1.0	x	x
**Lipid**						
(Z)-3-Oxo-2-(2-pentenyl)-1-cyclopenteneacetic acid	$C_{12}H_{16}O_3$	209.1172	209.1172	0.0	x	x
Jasmonic acid	$C_{12}H_{18}O_3$	211.1333	211.1329	2.0	x	x
12-Hydroxyjasmonic acid	$C_{12}H_{18}O_4$	227.1272	227.1278	-2.6	x	x
Plastoquinol-1	$C_{13}H_{18}O_2$	207.1378	207.1380	-0.8	x	x
Methyl jasmonate	$C_{13}H_{20}O_3$	225.1487	225.1485	0.8	x	x
(+)-7-Isomethyljasmonate	$C_{13}H_{20}O_3$	225.1486	225.1485	0.4	x	x
Isanic acid	$C_{18}H_{26}O_2$	275.2007	275.2006	0.4	x	x
Stearidonic acid	$C_{18}H_{28}O_2$	277.2164	277.2162	0.7	x	x
Gamolenic acid	$C_{18}H_{30}O_2$	279.2318	279.2319	-0.4	x	x
(9Z)-(13S)-12,13-Epoxyoctadeca-9,11,15-trienoate	$C_{18}H_{28}O_3$	293.2119	293.2111	2.7	x	x
(15Z)-12-Oxophyto-10,15-dienoic acid	$C_{18}H_{28}O_3$	293.2114	293.2111	1.0	x	x
Tuberonic acid glucoside	$C_{18}H_{28}O_9$	389.1804	389.1806	-0.5	x	x
8-[(1R,2R)-3-Oxo-2-{(Z)-pent-2-enyl}cyclopentyl]octanoate	$C_{18}H_{30}O_3$	295.2278	295.2268	3.5	x	x
(9Z,12Z,15Z)-Octadecatrienoic acid	$C_{18}H_{30}O_2$	279.2318	279.2319	-0.4	x	x
(9Z,11E,15Z)-(13S)-13-Hydroperoxyoctadeca-9,11,15-trienoic acid	$C_{18}H_{30}O_4$	311.2216	311.2216	0.0	x	x
(10E,12Z)-9-Oxooctadeca-10,12-dienoic acid	$C_{18}H_{30}O_3$	295.2268	295.2268	0.0	x	x
(9Z,11E)-13-Oxooctadeca-9,11-dienoic acid	$C_{18}H_{30}O_3$	295.2269	295.2268	0.3	x	x
(9Z,11E)-(13S)-13-Hydroxyoctadeca-9,11-dienoic acid	$C_{18}H_{32}O_3$	297.2420	297.2424	-1.4		x
Physcion 8-glucoside	$C_{22}H_{22}O_{10}$	447.1292	447.1286	1.4		x

Lysophosphatidic acid 0:0/18:2(9Z,12Z)	$C_{21}H_{39}O_7P$	435.2492	435.2506	-3.2	x	x
Phosphatidylethanolamine (0:0/16:0)	$C_{21}H_{44}NO_7P$	454.2924	454.2928	-0.9	x	x
Lysophosphatidylethanolamine(18:2/0:0)	$C_{23}H_{44}NO_7P$	478.2924	478.2928	-0.8	x	x
**Terpenoid**						
Azukisaponin I	$C_{42}H_{68}O_{13}$	781.4720	781.4733	-1.6		
Soyasaponin III	$C_{42}H_{68}O_{14}$	797.4662	797.4682	-2.5	x	x
Soyasaponin II	$C_{46}H_{76}O_{17}$	913.5137	913.5155	-2.0	x	x
Sandosaponin A	$C_{48}H_{76}O_{19}$	957.5029	957.5053	-2.5	x	
Phaseoside IV	$C_{48}H_{76}O_{17}$	925.5149	925.5155	-0.7	x	x
Dehydrosoyasaponin I	$C_{48}H_{76}O_{18}$	941.5096	941.5104	-0.9	x	x
Kaikasaponin II	$C_{48}H_{78}O_{17}$	927.5304	927.5312	-0.9	x	x
Soyasaponin I	$C_{48}H_{78}O_{18}$	943.5246	943.5261	-1.6	x	x
Asiaticoside	$C_{48}H_{78}O_{19}$	959.5195	959.5210	-1.6	x	x
Abrisaponin I	$C_{48}H_{74}O_{20}$	971.4831	971.4846	-1.6	x	x
**Chlorophyll**						
Pheophytin A	$C_{55}H_{74}N_4O_5$	871.5710	871.5732	-2.5	x	
Pheophytin B	$C_{55}H_{72}N_4O_6$	885.5501	885.5524	-2.6	x	x
